# Prognostic Factors Affecting Survival after Pulmonary Resection of Metastatic Renal Cell Carcinoma: A Multicenter Experience

**DOI:** 10.3390/cancers13133258

**Published:** 2021-06-29

**Authors:** Elisa Meacci, Dania Nachira, Edoardo Zanfrini, Jessica Evangelista, Elizabeth Katherine Anna Triumbari, Maria Teresa Congedo, Leonardo Petracca Ciavarella, Marco Chiappetta, Maria Letizia Vita, Giovanni Schinzari, Ernesto Rossi, Giampaolo Tortora, Marco Lucchi, Marcello Ambrogi, Fabrizia Calabrò, Francesco Petrella, Lorenzo Spaggiari, Marco Mammana, Andrea Lloret Madrid, Federico Rea, Diomira Tabacco, Stefano Margaritora

**Affiliations:** 1 Department of General Thoracic Surgery, Fondazione Policlinico Universitario “A. Gemelli”, IRCCS, Università Cattolica del Sacro Cuore, 00167 Rome, Italy; edoardo.zanfrini@policlinicogemelli.it (E.Z.); jessicaevangelista@policlinicogemelli.it (J.E.); mariateresa.congedo@policlinicogemelli.it (M.T.C.); leonardo.petraccaciavarella@policlinicogemelli.it (L.P.C.); marco.chiappetta@policlinicogemelli.it (M.C.); marialetizia.vita@policlinicogemelli.it (M.L.V.); diomiratabacco@policlinicogemelli.it (D.T.); stefano.margaritora@unicatt.it (S.M.); 2 Section of Nuclear Medicine, Department of Radiological Sciences and Haematology, Università Cattolica del Sacro Cuore, 00167 Rome, Italy; elizakat@virgilio.it; 3 Department of Medical Oncology, Fondazione Policlinico Universitario “A. Gemelli”, IRCCS, Università Cattolica del Sacro Cuore, 00167 Rome, Italy; giovanni.schinzari@policlinicogemelli.it (G.S.); ernesto.rossi@policlinicogemelli.it (E.R.); giampaolo.tortora@unicatt.it (G.T.); 4 Department of General Thoracic Surgery, Azienda Ospedaliero Universitaria Pisana, 56124 Pisa, Italy; Marco.lucchi@unipi.it (M.L.); marcello.ambrogi@unipi.it (M.A.); fabrizia.calabro@unipi.it (F.C.); 5 Department of Thoracic Surgery, IEO European Institute of Oncology IRCCS, 20141 Milan, Italy; francesco.petrella@ieo.it (F.P.); lorenzo.spaggiari@ieo.it (L.S.); 6 Department of Oncology and Hemato-Oncology, Università degli Studi di Milano, 20141 Milan, Italy; 7 Thoracic Surgery Division, Department of Cardiac, Thoracic, Vascular Sciences and Public Health, Padova University Hospital, 35122 Padova, Italy; marcomammana@unipd.it (M.M.); alloret@libero.it (A.L.M.); Federico.rea@unipd.it (F.R.)

**Keywords:** renal cell carcinoma, lung metastases, metastasectomy, metastatic renal cell carcinoma

## Abstract

**Simple Summary:**

This multicentric paper aimed at evaluating the role of pulmonary metastasectomy in patients affected by metastatic renal cell carcinoma. The impact of pulmonary metastasectomy was analysed with respect to long-term survival and disease-free survival in a wide population of patients affected by pulmonary metastases from renal cell carcinoma. The prognostic value of factors affecting survival, disease-free interval and disease-free survival was evaluated. Our results aid clinicians in identifying those patients affected by pulmonary metastases from renal cell carcinoma who are more likely to benefit from pulmonary metastasectomy.

**Abstract:**

In this paper we aimed to address the role of pulmonary metastasectomy (PM) in patients affected by Lung Metastases (LM) from Renal Cell Carcinoma (RCC) and to analyse prognostic factors affecting overall survival (OS), disease-free interval (DFI) between primary RCC and first LM, and disease-free survival (DFS) after PM and before lung recurrence. Medical records of 210 patients who underwent PM from RCC in 4 Italian Thoracic Centres, from January 2000 to September 2019, were collected and analysed. All patients underwent RCC resection before lung surgery. The main RCC histology was clear cells (188, 89.5%). The 5- and 10-year OS from the first lung operation were 60% and 34%, respectively. LM synchronous with RCC (*p* = 0.01) and (Karnofsky Performance Status Scale) KPSS < 80% (*p* < 0.001) negatively influenced OS. Five- and 10-year DFI were 54% and 28%, respectively. The main factors negatively influencing DFI were: male gender (*p* = 0.039), KPSS < 80% (*p* = 0.009) and lactate dehydrogenase > 1.5 times 140 U/L (*p* = 0.001). Five- and 10-year disease-free survival were 54% and 28%, respectively; multiple LM (*p* = 0.036), KPSS < 80% (*p* = 0.001) and histology of RCC other than clear cells negatively influenced disease-free survival. Conclusions: patients with KPSS > 80%, single metachronous LM with a long DFI from RCC diagnosis, and clear cell histology, benefit from pulmonary metastasectomy.

## 1. Introduction

Renal cell carcinoma (RCC) is the seventh most frequently diagnosed cancer worldwide, accounting for about 3% of all cancers in adults. It represents the third most common urinary tract malignancy [1], with more than 140,000 deaths in 2013 [2]. About 30% of patients affected by RCC show distant metastases at the time of diagnosis, while another 25% develop metachronous metastases [3]. The lack of an effective therapy for advanced disease leads to RCC being the sixth leading cause of death for cancer worldwide [4]. Taking into account that the 5-year survival rate of patients with untreated metastatic disease ranges from 0% to 18% [5], the strategy to handle this particular group of patients needs to be addressed. The recent introduction of systemic therapies based on Vascular Endothelial Growth Factor (VEGF) and mammalian Target of Rapamycin (mTOR) targets and immune checkpoint inhibitors has improved the prognosis of metastatic patients, with a median overall survival (OS) reported between 26.4 and 32.0 months [6,7]. However, the effect of these drugs is to reduce the tumour size by 20–30%, improving progression-free survival and OS, without complete eradication of the disease [8,9]. The foremost metastatic site of RCC is the lung, which is involved in 45–75% of metastatic cases [10]. To date, the most applicable management of RCC patients with lung metastases is a longstanding debate. Durable complete responses are achieved in less than 10% of patients treated with immunotherapies (interferon alpha and interleukin-2), and outcomes have only slightly improved [11], while severe therapy-related toxic effects reach high levels [12]. Complete responses remain scarce and there are still some treatment-related adverse events also in patients treated with agents targeting the VEGF/PDGFR/mTOR pathway [13]. The median OS in these patients amounts to about 22 months [14].

Despite the fact that the presence of RCC-derived lung metastases (LM) is usually considered as a systemic disease, since the first successful resection of an RCC LM in 1939 [15], surgical eradication of LM from RCC has gained popularity. Many observational studies have shown survival benefits offered by an aggressive surgical approach, reporting 5-year survival rates ranging from 18% to 75% [16]. On this basis, although no randomized clinical trials have established the role of surgical metastasectomy, in the National Comprehensive Cancer Network (NCCN) clinical practice guidelines of RCC (version 4.2018, accessed on 23 April 2018), RCC with lung metastases is classified as stage IV disease, with nephrectomy and resection of lung metastases being the first-line therapy.

Many studies evaluating the impact of pulmonary metastasectomy vs. conservative treatment in patients affected by isolated LM demonstrated that surgical resection offers clear advantages in terms of OS [17,18].

Consequently, surgery should be the preferred choice for eligible patients with potentially completely resectable LM. However, the remarkably good results showed by surgical resection are probably affected by an unavoidable selection bias regarding patients undergoing surgical metastasectomy (SM), as those referred to surgery are patients with more localized disease and better performance status. Some prognostic scoring systems have been reported and have evolved over the years in order to better select patients for surgery [19,20,21] but, currently, no definitive guidelines are available to select patients for SM. Further prognostic factors need to be identified to properly select patients who can take real advantage from SM. Several previous studies are single-centred, based on a small sample size and without long-term follow-up. The lack of large-sample-sized research evidence led to difficulties in the definition and selection of indications for pulmonary metastasectomy (PM) in RCC patients.

Therefore, we developed a multicentric retrospective study to evaluate the clinicopathological variables affecting OS, Disease-Free Interval (DFI) and Disease-Free Survival (DFS) in patients who underwent surgical resection of LM from RCC.

## 2. Materials and Methods

Medical records of 210 patients who underwent PM for primary RCC in 4 Italian Thoracic Centres, from January 2000 to September 2019, were collected.

After approval of this study by our Internal Review Board, all clinical data and surgical reports of patients were retrospectively analysed. The main clinicopathological variables reviewed were: gender, age, KPSS, blood test values, primary cancer histology, side of kidney tumour, neoadjuvant and adjuvant therapies for renal cancer, date of nephrectomy and lung metastasectomy, DFI (time between nephrectomy and first pulmonary metastasis diagnosis), characteristics of lung metastases (number, side, dimensions, type of resection, etc.), date of last follow-up (FUP), patient status at FUP, adjuvant therapy after PM, reiterative lung metastasectomies. Age was classified as younger and older than 50 years old; patients were divided into two groups according to KPSS > 80 or <80%; subtypes of RCCs were noted as clear cell, papillary, chromophobe and others, depending on the analysed pathological reports.

Characteristics of lung metastases were analysed as follows: single vs. multiple (≥2) LM; dimensions of LM < 3 cm vs. ≥3 cm; synchronous vs. metachronous metastases. OS was defined as time interval between the first pulmonary metastasectomy and the last FUP date or death. DFS was defined as the time interval between PM and the date of pulmonary recurrence.

The primary renal tumour was under control in all patients and no other metastatic site was documented during preoperative examinations before pulmonary metastasectomy. No patient underwent induction therapy before renal surgery. In all cases of new pulmonary metastases occurrence (single or multiple), lung metastasectomy was performed when technically and functionally feasible.

### 2.1. Preoperative Assessment

Before PM, all patients were evaluated by routine blood tests, electrocardiography (or other cardiological second level tests, when necessary), pulmonary function test, total body computed tomography (CT), brain CT and bone scintigraphy.

Lung resection was performed under general anaesthesia and single-lung ventilation. A wedge or a major anatomic resection (in case of central or big lesions) with macroscopic tumour-free borders was performed by thoracotomy or Video-Assisted Thoracic Surgery (VATS), according to the operation period or Centres’ surgical preferences.

### 2.2. Statistical Analysis

Continuous variables were expressed as mean ± standard deviation, categorical ones as percentage.

Survival and disease-free interval analyses were performed by Kaplan–Meier method and the principal factors affecting survival and recurrence were investigated by Log-Rank tests. All covariates with *p* < 0.2 were selected for Cox proportional hazards regression model to assess prognostic independent factors. For all tests, a *p*-value < 0.05 was considered as statistically significant. Statistical analysis was performed using IBM SPSS Statistics for Macintosh (version 25.0, IBM Corp, Armonk, NY, USA).

## 3. Results

### 3.1. Clinical Characteristics

The main clinical characteristics of the 210 patients analysed are reported in Table 1. The population was composed of 169 males (80.5%) and the mean age was 64.28 ± 9.70 years (range: 36–83). All patients underwent renal tumour resection before lung surgery. The main renal cancer histology was clear cells (188, 89.5%), followed by papillary (5, 2.4%), chromophobe (4, 1.9%) and other types (13, 6.2%).

At the time of renal cancer diagnosis, 31 patients (14.8%) had a synchronous lung metastasis. Thirty-five patients (13.9%) underwent two consecutive lung resections (22 for bilateral metastases and 13 for ipsilateral recurrence); the DFI between first lung metastasectomy and ipsilateral metastasis recurrence was 24.14 ± 33.61 months, and the mean time between the first two lung surgeries was 12.75 ± 15.68 months. Eight patients (3.8%) underwent three lung operations and 5 (2.4%) four lung re-operations in a mean time of 153.22 ± 35.47 months (range: 110–195). No patient underwent neoadjuvant therapy before pulmonary surgery.

The mean size of lung metastases was 2.09 ± 1.80 cm (range: 1–13), with a mean number of 2.08 ± 2.01 lesions (range: 1–13) per patient. Eighty patients (38.8%) had multiple lung metastases; metastases were located on the right side in 124 cases (59%), predominantly in the lower lobes (123 cases, 58.6%).

When technically feasible, the principal type of lung metastasectomy was a wedge resection (163 cases, 77.6%). Thirty-day mortality was null. After lung surgery, 24 patients (11.4%) underwent traditional radio-chemotherapy and 6 (2.8%) immunotherapy (4 checkpoint inhibitors and 2 Interferon).

### 3.2. OS and DFI

During a mean FUP of 109.62 ± 86.71 months (Median: 87.11) from the first operation for renal cancer, 71 patients (33.80) had died (62 because of renal cancer disease). The 5- and 10- year OS from the first lung operation was 60% and 34%, respectively. In particular, when evaluating the OS in cohorts of patients treated before 2007 (pre-Tyrosine Kinase Inhibitors—TKI—era) and after 2007 (TKI era), no difference was found (5-year survival: 56% vs. 70%, *p* = 0.490). The mean DFI was 60.73 ± 67.54 months.

### 3.3. Prognostic Factors

**Figure 1:**
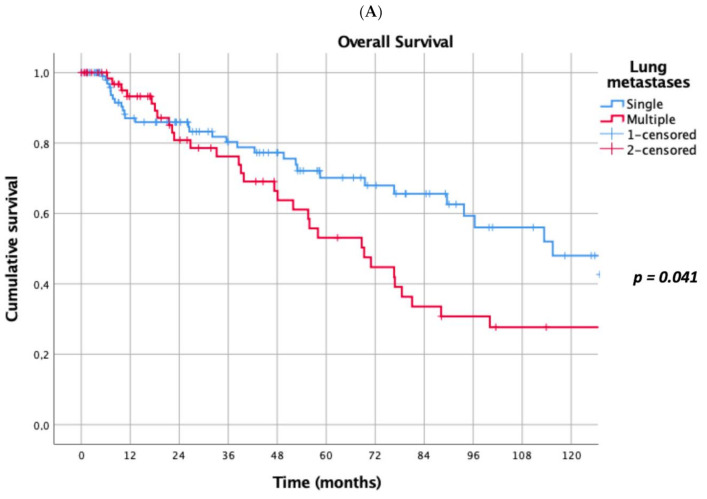

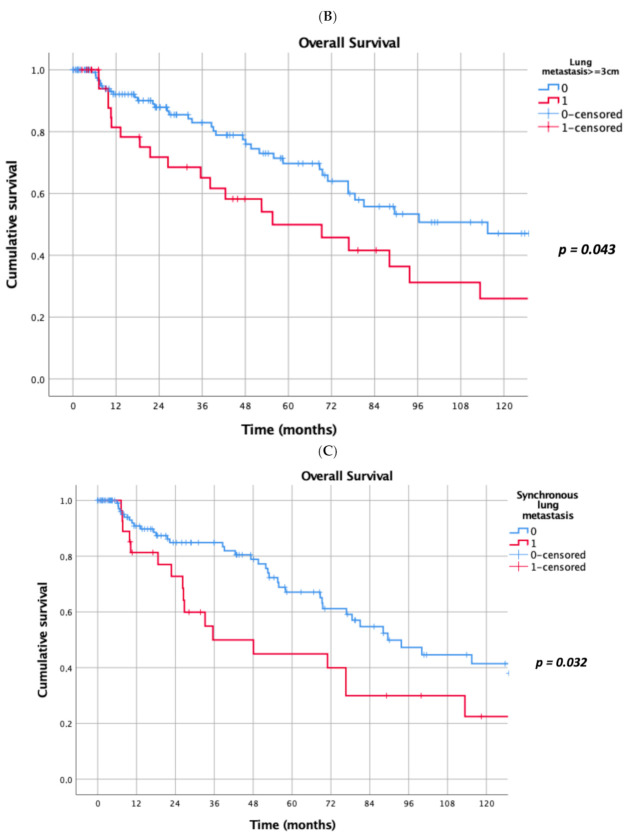

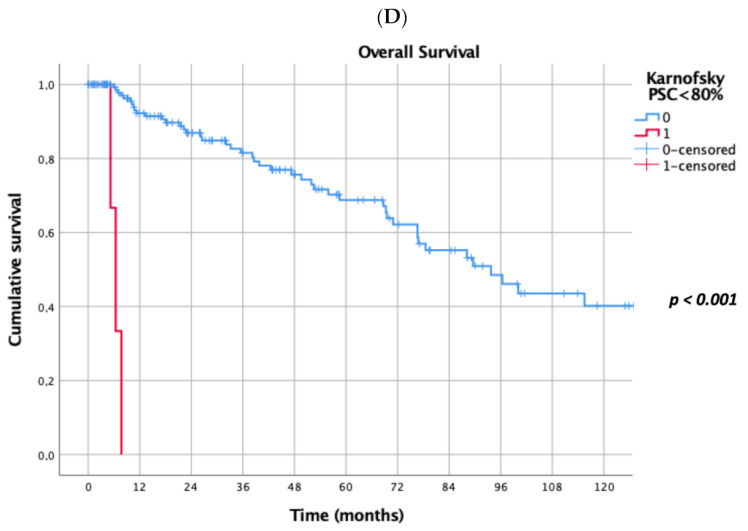
(**A**): Prognostic factors negatively influencing Overall Survival (OS) at univariate analysis: presence of multiple lung metastases. (**B**): Prognostic factors negatively influencing OS at univariate analysis: dimensions of lung metastasis ≥ 3 cm. (**C**): Prognostic factors negatively influencing OS at univariate analysis: lung metastasis synchronous with primary renal cancer. (**D**): Prognostic factors negatively influencing OS at univariate analysis: Karnofsky Performance Status Scale (KPSS) < 80%.

Prognostic factors negatively influencing OS at univariate analysis were: presence of multiple (≥2) lung metastases (5-year survival 45% vs. 69%, *p* = 0.041, Figure 1A), dimensions of lung metastases ≥3 cm (5-year survival 44% vs. 65%, *p* = 0.043, Figure 1B), lung metastases synchronous with primary renal cancer (5-year survival 40% vs. 62%, *p* = 0.032, Figure 1C), KPSS < 80% (5-year survival 0% vs. 63%, *p* < 0.001, Figure 1D); adjuvant therapies after lung metastasectomy had only a trend towards significance (*p* = 0.069). At multivariate Cox regression analysis, the confirmed independent risk factors for worse survival were: synchronous metastasis (*p* = 0.008) and KPSS (*p* < 0.001), (Table 2).

## 4. Discussion

Investigating the main variables affecting DFI between primary renal cancer and first pulmonary metastasis, only male gender, KPSS < 80% and lactate dehydrogenase > 1.5 times the upper limit of normal range (140 U/L) resulted in significant independent risk factors both in univariate and multivariate analyses (Table 3).

Five- and 10-year disease-free survival after pulmonary metastasectomy and before lung disease recurrence was 54% and 28%, respectively. The main factors negatively influencing disease recurrence at univariate analyses and confirmed at Cox regression analysis were: multiple lung metastases, KPSS < 80% and histology of primary kidney cancer other than clear cells (Table 4).

This study retrospectively analysed the objective response rate, represented by OS, DFI, DFS and prognostic factors, in 210 patients who underwent intentional curative resection of LM from metastatic RCC.

Five- and 10-year OS from the first lung operation were 60% and 34%, respectively, in our multicentric experience. Our data are in line with the results recently published by Saricam [22], who reported a 5-year survival rate of 62.5%, but exceed the outcomes of surgery described in other previous studies [23,24] and meta-analysis [19], where 5- and 10-year OS rates were 43% and 20%, respectively.

In 2017, we already reported our monocentric experience with a very high 5- and 10-year OS of 75% and 59%, respectively [25]. This occurrence may result from a careful selection of patients with a very good performance status and limited disease, likely to be completely resectable, and the exclusion of patients who did not meet criteria for surgery. Additionally, surgical techniques and recent progresses in perioperative management allow better prognoses, as demonstrated by relatively low morbidity and mortality, with complications in <15% of cases and no mortality in ours as in most studies [19]. Only 31 patients (14.8%) enrolled in the present study showed pulmonary synchronous RCC metastases and 48 (22.8%) were operated on more than one time (from 2 to 4 times). Remarkably, 61.9% (130) of patients underwent surgery for single pulmonary metastasis. This percentage of single LM patients is very high when compared with other studies (47–56%) [26,27,28] and, in addition, more than 89% of patients were affected by LM from clear cell RCC, which is usually accepted as a significant favourable prognostic factor in patients who underwent PM [29]. No patients included in our experience underwent alternative therapies or incomplete resection for diagnostic purposes. CT scans were carefully analysed in order to evaluate information about radical resectability of the nodules and to predict incomplete resection. Partial resection, in fact, significantly influences the 5-year survival rate that reaches 44% in case of complete resection, while it is only 14% for incomplete resection and 11% for no surgical resection [5,30].

Comparing the outcome of patients affected by single vs. multiple LM, we found a statistically significant better OS in patients surgically treated for a single LM (5-year survival of 69%) when matched with multiple LM patients (5-year survival of 45%). This result is in accordance with previous studies [22,30]. Many authors reported that a worse prognosis is combined with the presence of multiple metastases: Hofmann et al. found a worse prognosis in patients with more than 6 metastases [20], while Saricam [22] suggested that more than three LM or lesions measuring more than 4 cm^3^ clearly predict a poor prognosis. We actually found a 44% 5-year survival vs. 65% (*p* < 0.043) in the case of metastases ≥3 cm in diameter, confirming that dimensions of LM influence OS, in accordance with many previous experiences [25,30,31].

The presence of lung metastasis synchronous with primary renal cancer (5-year survival 40% vs. 62%, *p* = 0.032, Figure 1C), and a KPSS < 80% (5-year survival 0% vs. 63%, *p* < 0.001, Figure 1D) revealed to be prognostic factors negatively influencing OS at multivariate analysis in our study. In a recent meta-analysis, Zhao et al. [30] reported that synchronous metastasis (HR 2.49, 95% CI 1.46–4.24, *p* < 0.001) and short DFI are predictors of poor survival after PM. Patients with a long DFI (more than 32 months) from RCC to the first pulmonary metastasis showed a better prognosis also in Saricam’s experience [22] as in many other experiences [5,18,20,26,32,33,34,35,36]. Meimarakis et al. [21] reported better OS in patients with metachronous disease when compared with synchronous disease {median OS 54.0 months (CI 95%, 30.3–77.7 months) and 28.2 months (CI 95%, 18.1–38.4 months) respectively; *p* = 0.18}. Luzzi et al. [31] in 2017 reported that at univariate analysis, the DFI had a significant impact on survival (5-year survival of 58% for patients with DFI  ≥  24 months vs. 46% in patients with DFI < 24 months; *p* =  0.048).

The main strength of our study is the large number of patients analysed, which is one of the biggest in literature. Limitations of this study are its retrospective design, the small number of patients with a KPSS < 80% and the high percentage of patients affected by single LM. Moreover, we were not able to define the significance of lymphadenectomy and adjuvant therapies after PM because data from different centres were not always available.

## 5. Conclusions

The present study was conducted with the aim to identify criteria for selecting patients with isolated LM who would benefit from surgical resection. Our results suggest that a KPSS < 80%, male gender, the presence of synchronous and multiple LM, the diameter of LM ≥ 3 cm, high levels of lactate dehydrogenase and histology of primary RCC other than clear cell carcinoma seem to be predictors of poor survival after PM.

Even though the presence of synchronous metastases worsens prognosis, it does not exclude surgery if radical resection can be obtained. Further studies evaluating the role of lymphadenectomy and adjuvant therapies in patients affected by isolated lung metastases from RCC are needed.

## Figures and Tables

**Table 1:** Clinicopathological characteristics of the patients.

Variables	#210 Patients
Age (years)	64.28 ± 9.70
Gender (male)	169 (80.5%)
KPSS < 80%	4 (1.9%)
Haemoglobin < lower limit of the normal range (Men: 13.5–17.5 g/dL, Women: 12.0–15.5 g/dL)	35 (16.7%)
Lactic dehydrogenase > 1.5 times the upper limit of normal range (140 U/L)	21 (10%)
Calcium > 10 mg/dL	25 (11.9%)
Neutrophils > upper limit of the normal range (2.0–7.0 × 10⁹/L)	18 (8.6%)
Platelets > upper limit of the normal range (150,000–400,000 cells/µL)	10 (4.8%)
Histology of renal cancer	
Clear cells	188 (89.5%)
Papillary	5 (2.4%)
Chromophobe	4 (1.9%)
Other	13 (6.2%)
Number of pulmonary metastases	Mean: 2.08 ± 2.01 Median: 1, range 1–13
Single vs. multiple metastases	130 (61.9%)/80 (38.1%)
Synchronous metastases	31 (14.8%)
Pulmonary side (right)	124 (59%)
Type of lung resection	
Wedge	163 (77.6%)
Segmentectomy/Lobectomy	47 (22.4%)
Dimensions of pulmonary metastases (cm)	Mean: 2.09 ± 1.80 Median: 1.80 range: 1–12.8
Localization of pulmonary metastases	
Upper lobe	110 (52.4%)
Median lobe	37 (17.6%)
Lower lobe	123 (58.6%)
Adjuvant therapy after metastasectomy	
CT	21 (10%)
RT	3 (1.4%)
Immunotherapy	6 (2.9%)

**Table 2:** Multivariate Cox regression analysis for negative prognostic factors for OS.

Variables	HR (95% CI)	*p*-Value
Synchronous metastasis	2.934 (1.324–6.505)	0.008
KPSS < 80%	24.381 (3.842–154.700)	<0.001

**Table 3:** Univariate and multivariate analyses for negative prognostic factors for DFI between primary renal cancer and first pulmonary metastasis.

Variables	Univariate Analysis	Multivariate Analysis	
	*p*-Value	HR (95% CI)	*p*-Value
Male Gender	0.048	1.899 (1.032–3.495)	0.039
KPSS < 80%	0.001	18.104 (2.090–156.849)	0.009
Lactate dehydrogenase >1.5 times 140 U/L	<0.001	3.385 (1.659–6.906)	0.001

**Table 4:** Univariate and multivariate analyses for negative prognostic factors for disease-free survival after pulmonary metastasectomy and before lung disease recurrence.

Variables	Univariate Analysis	Multivariate Analysis	
	*p*-Value	HR (95% CI)	*p*-Value
Histology of primary renal cancer (other than clear cell)	0.036	3.476 (1.239–9.748)	0.018
Multiple lung metastases	0.028	1.721 (1.036–2.858)	0.036
KPSS < 80%	0.006	15.649 (2.992–81.851)	0.001

## Data Availability

The datasets used and/or analysed during the current study are available from the corresponding author on reasonable request.
